# A sanctuary: Mourning the loss of the classroom during COVID

**DOI:** 10.1177/1473325020981084

**Published:** 2021-03

**Authors:** Lakindra Mitchell Dove

**Affiliations:** Portland State University, USA

**Keywords:** Resilience, trauma, students, remote learning, Covid-19, trauma-informed pedagogy

## Abstract

This reflexive essay explores the challenges and successes that I encountered as a professor during the transition from face-to-face teaching to remote teaching due to COVID. The essay outlines my thought processes and emotional responses to how unfamiliarity with teaching remotely, coupled with the stress of a pandemic, significantly impacted my teaching style. It also highlights my observations of students’ experiences from their shared discussions and interactions with other students as they navigated the initial onset of challenges during the spring term of 2020. The essay discusses the importance of adaptability during a time when we were collectively experiencing trauma, and embracing teaching practices that were supportive of students' educational and emotional needs. It highlights how navigating these challenges were beneficial for both myself and students, as we collectively addressed the complexities of changes to our personal and academic lives. This essay includes lessons learned as a professor and how I was able to maintain the integrity of the classroom by holding space for the experiences of students during a pandemic.

On March 12, 2020 there was a sense of heaviness present. The day felt extremely tense. It also coincided with the last day of winter term, so feelings of stress and anxiety were to be expected, but there was something unusual about this energy. We would soon discover that it was the precursor to loss. As information regarding COVID increased, and the seriousness of the situation heightened, I shifted gears and intuitively started to prepare students for the unknown. Initially I was all set to wrap up the term, ending social work practice with individuals and families by recapping salient points and highlighting key information. This course was particularly challenging for this cohort of students as I pushed them to be vulnerable, take risks, and use critical thinking skills. They were playing it safe, too concerned about writing a perfect biopsychosocial-spiritual assessment. I questioned whether I was holding them to high standards, expecting them to know how to write clinically. I reminded myself of what I witnessed while practicing in the field, the multiple assessments and case notes that I read, revealing one of the reasons why I decided to transition from direct practice to academia. I was here to inspire the next generation of social workers. I was equipped with years of practice experience and recognized the importance of sound social work practice skills. I knew they were capable of rising to the challenge, and they did.

As the day unfolded, I sensed a subtle look of fear in the eyes of students. It was as if they were scanning the room for comfort and familiarity, perhaps some instruction about what to do with their sudden and unexpected emotions. I suspected that they too sensed the heaviness in the air. The looming energy of COVID. The university had not made any official announcements, but there were rumblings among faculty, staff, and students. In the copier room, the hallways, and offices. We made speculations about the potential impacts of COVID, this strange thing that we were not familiar with, yet it quickly made its presence known in our lives. The first wave of information started with the recommendation that individuals who were ill or had a compromised immune system, stay home. Students came into my office that day confused about whether they should remain at school or go home. They were perplexed by the information, but also concerned about their well-being. One student thanked me for a great term and apologized for not being able to fully show up as they normally had as a student. They shared their journey of coming to terms with a recent medical diagnosis. I assured them that there was no need to apologize and strongly suggested they prioritize safety and well-being.

Stepping into the final class of the day felt overwhelming. It was like students were waiting for someone to let them in on a secret, but faculty, just like students, remained in the dark. I was prepared to lecture, but my instincts and many years of practicing in the field, guided me to remove my professor hat and exchange it for my social worker hat. Students were not in a space to consume more content. It was the end of the term, tensions were high, and conversations about COVID were prioritized. Students needed me to demonstrate direct service skills, those same skills that we discussed in class throughout the term. I conducted an “assessment” of the classroom (reading non-verbal cues and body language) and facilitated a discussion about what I sensed from students. They shared that they were already losing their jobs, specifically those in the service industry. Some students had family members who were out of the country in areas already impacted by the virus and many worried about the unknown. There is something to be said about professors who are able to make space for uncertainty and demonstrate vulnerability. It was important for students to feel comfortable expressing their initial reactions in a setting that felt supportive. Parker et al. (2010: 91) note, “by welcoming the whole student into our classes, unfamiliar aspects of who they are and what they care about suddenly comes into view.” This is exactly what unfolded that day in the classroom.

I was not expecting students to share such immediate impacts of COVID. At that time, we had no idea what we were facing and how we would be impacted for months to come. As stated by hooks (1994) to teach in a manner that responds and cares for the souls of our students is essential if we are to provide the necessary conditions where learning can most deeply and intimately begin. This was brought to my attention by a student who encountered difficulty completing a final assignment and requested an extension after the due date. I recognized that this was not the time to be punitive or use this situation as a teachable moment in being responsible. This student needed empathy and compassion, conveyed through acknowledgement that the world was changing right before our eyes. The last thing that any student should have been concerned about was due dates or a professor’s unwillingness to be flexible. Instead of setting any parameters for submitting the assignment, I negotiated by taking the student’s needs into consideration. A few days later I received the assignment via email with this message included, “I appreciate you and your ability to recognize when your students need support and/or understanding. That isn’t something that happens very often and I admire how you truly model trauma-informed practices in your teaching and in the classroom. It does not go unnoticed. Thank you.” This student also shared how they experienced homelessness, and had recently secured housing, which was now disrupted by the pandemic. They had to book a hotel for the night and planned to return to couch surfing with friends. I wept. It was the culmination of tension, anxiety, and uncertainty trapped inside of my body that activated this response. I could no longer carry the burden. I knew students would be disproportionately impacted by COVID, but I underestimated the swiftness and severity of disruption and devastation. This vulnerable and courageous student provided a glimpse of students’ experiences that shifted my perspective. I also wept because the sanctuary, the classroom as we once knew it, no longer existed. On March 18, 2020 the university announced plans to shift to remote learning for the duration of spring term. There would be no opportunity to physically connect with graduating students in their final term of the BSW Program. This was heartbreaking.

As I leaned heavily on my self-care practices for sustainability during such uncertainty, I reflected on all of the times that I complained about students – a jammed packed schedule, teaching back to back classes, and being “on,” a performative act. I complained about how to do it well week after week and attend to other responsibilities. Now a major component, the classroom, was stripped away and I felt lost. How was I supposed to be and do as I normally would in a virtual setting? How would I foster a sense of community? How would I create a brave space for students to share their experiences, ideas, and opinions? How would I accomplish all of this without the sanctuary? Through mourning the loss of the classroom, I expressed gratitude and acknowledged how I had taken the classroom for granted. I had minimized its value and worth, and now I was forced to teach without it. I pondered if it was possible to recreate the classroom in a virtual space. I was doubtful, but open to trying something new. I was also willing to be vulnerable and step outside of my comfort zone.

Initial excitement about exploring something new carried me for a few weeks, then I hit a wall. By week three my energy was drastically low and I was exhausted. The adrenaline wore off and reality set in. This too felt familiar, I was processing another wave of trauma. The signs were there. I was unmotivated, had difficulty concentrating, and I was not interested in teaching. I questioned what I was doing and why. I realized that I had attempted to maintain a teaching style that I was accustomed to while in the classroom, and it would be impossible to implement in this new space of remote teaching. The excitement of trying something new vanished. I achieved the initial task of tackling remote teaching. I could log on, students could log on, I knew how to operate video and audio features, and students could see my shared screen. I accomplished the basics, but the monotony of zooming from week to week contributed significantly to my downward spiral. I was exhausted and missing rich discussions, expressions of thoughts and emotions, and the overall vibe of the classroom. There were seven weeks remaining in the term and I did not know how much longer I could sustain in a space that felt so sterile. I was committed to making the most of the experience for myself and graduating students. This was our final class together and even if it was not ideal, we would make it memorable. The remainder of the term was met with highs and lows, but I embraced those moments, knowing they were valuable experiences during a time when no one could have ever imagined a massive pivot to remote learning with minimal instruction and limited time, while grieving the loss of a sanctuary.

There were many areas of growth throughout that time. This growth was disguised by the challenge to remain calm and collected during the transition. I was determined to get to the end of the term without completely breaking down. I learned that routines are great, but it is reasonable to expect changes, even if changes are precipitated by unforeseen circumstances. There was a fair amount of resistance to the transition to remote learning and teaching. This resistance stemmed largely from fear of the unknown. None of us signed up for this, was a reverberating phrase around higher education institutions. There were so many assumptions that remote learning was equivalent to online learning. They are not one in the same. What we did was apply a band-aid to a severe hemorrhage that needed critical attention. The shift to remote learning was a knee jerk reaction and an attempt to ameliorate a dire situation. Resistance caused further damage and flexibility was warranted. I learned so much about myself and my ability to adapt in such a short period of time. I was equally impressed by the adaptational skills displayed by students. I have gained a new respect for standards. Standards are ideal, but should be modified. I had to come to grips with underlying perfectionist tendencies and there were times when they got the best of me. I reminded myself each week that I was doing the best that I could in the moment, and that going with the flow was in my best interest and that of students. My commitment to creating robust assignments and delivering flawless lectures was unrealistic. Synchronous sessions were optional and students were not penalized if they were not able to attend. Surprisingly, the majority of students logged on each week and remained for the entire session. I told students that they would have to work hard to fail the course and I was very intentional about streamlining content and coursework. I abandoned these ideals and carved out time for students to chat in small groups (breakout sessions) about whatever they wanted to discuss, just as they would when gathering before class. This seemed to boost morale, as students returned to the main session smiling and in good spirits. We all had taken for granted the importance of these seemingly small interactions.

Students met me where I was and I did the same. I never imagined that I would share such intimate aspects of my life with students, but they appreciated seeing me model parenting, while simultaneously holding class sessions. Students saw me in a different element, one that challenged preconceived beliefs about me as a professor and allowed them to see me as a human being and a mother. In exchange, I enjoyed seeing students in different roles. We all adjusted to how our home and work/school life collided while quarantined. In this new way of engaging, we discovered that learning is not restricted to course content. It is important to honor the value of lived and shared experiences in the classroom. This was most apparent during a pandemic. Students needed the opportunity to connect with one another on a level that was not limited to enforcing learning objectives of a course. Learning is also appealing to the social and emotional needs of students within the context of a classroom setting. Students were appreciative of opportunities to slow down, discuss the present moment, and hear how others were impacted by COVID. In this way we are able to capture the essence of humanity in a virtual setting. As we neared the end of the term and students delivered final presentations, I was struck by how I was emotionally moved. I felt present, engaged, and connected in those moments. Through individual manifestos and group projects, students displayed the ability to dig deep, work collaboratively, and produce meaningful and thought-provoking content. Although the end of the year is prone to emotional overload, this was different. Connection is something that we struggled with and towards the end, we were able to collectively come together. We embodied the essence of human connection and figured out how to convey this through computer screens.

As the future remains unknown, many colleges and universities have decided to begin fall term remotely. Many faculty and students returned to the classroom with a better understanding of the virtual environment, while also acknowledging that it is not the same and could never replace the experience of an in-person class. As a professor, I have a new found sense of confidence in my ability to adapt and do things differently. I feel that I have been well prepared through trial and error to tackle remote teaching with confidence. I am also equipped with strategies to foster a sense of community virtually. I am certain that despite whether gathered physically in a classroom or not, I will be able to provide a sanctuary for students to learn, connect, and grow. I am thankful for the challenging experiences. They have significantly shaped my approach to teaching and learning and will have a lasting impact on how I navigate future challenges.
